# Structural and Functional Picosecond Laser Modification of the Nimonic 263 Superalloy in Different Environmental Conditions and Optimization of the Irradiation Process

**DOI:** 10.3390/ma16031021

**Published:** 2023-01-22

**Authors:** Boris Rajčić, Tatjana Šibalija, Vladimir Nikolić, Miha Čekada, Jelena Savović, Sanja Petronić, Dubravka Milovanović

**Affiliations:** 1 Institute of General and Physical Chemistry, 11158 Belgrade, Serbia; 2 Faculty of Information Technology, Belgrade Metropolitan University, 11158 Belgrade, Serbia; 3 Jozef Stefan Institute, 1000 Ljubljana, Slovenia; 4 Vinca Institute of Nuclear Sciences, University of Belgrade, 11351 Belgrade, Serbia

**Keywords:** Nimonic 263, picosecond laser, surface modification, argon-rich atmosphere, nitrogen-rich atmosphere, Taguchi’s robust parameter design

## Abstract

In this experimental study, picosecond laser treatment was performed on a nickel-based superalloy Nimonic 263, aiming to investigate the surface effects induced by irradiation in different atmospheric conditions and, concerning changes in surface composition, regarding the possibility for improvement of its functionality. Besides the varying laser parameters, such as a number of pulses and pulse energy, environmental conditions are also varied. All surface modifications were carried out in standard laboratory conditions and a nitrogen- and argon-rich atmosphere. The resulting topography effects depend on the specific laser treatment and could be categorized as increased roughness, crater formation, and formation of the laser-induced periodic surface structures (LIPSS). Changes in the chemical surface composition are distinguished as the potential formation of the protective oxides/nitrides on the sample surface. Numerous characterization techniques analyse the resulting effects on the topography and surface parameters. The multi-response parametric optimization of the picosecond laser process was performed using an advanced statistical method based on Taguchi’s robust parameter design. Finally, the optimal parameter conditions for Nimonic 263 modification are suggested.

## 1. Introduction

Nickel-based superalloys maintain their mechanical strength, resistance to thermal deformation, surface stability, corrosion, and oxidation resistance at high temperatures [1,2,3]. Depending on the chemical composition, certain superalloys can withstand temperatures above 1200 °C [4]. Due to these properties, nickel-based superalloys are currently most used in aircraft turbine components, steam turbine power plants, nuclear power plants, space vehicles, rocket engines, and medicine [5,6,7,8]. More precisely, components made of Nimonic 263 superalloy can sustain extreme and rigorous operating conditions without yielding [9]. However, this advantage makes Nimonic 263 material difficult to process, micromachining, and improve by surface treatments.

Laser structuring is proven as a precise and controllable way to enhance the properties and functionality of metallic surfaces by increasing the strength, active surface, and biocompatibility of alloys. It is a successful tool for improving the hardness of surfaces susceptible to surface stress [10] or altering the absorption properties and biocompatibility by laser-induced periodic surface structures [11,12]. The interaction between pulse laser irradiation and metal surfaces is a complex process with optical, physicochemical, and mechanical changes which depend on laser parameters such as pulse duration, laser energy, wavelength, and laser frequency [13]. Surface characteristics and parameters, the melting temperature, material roughness, and laser irradiation absorption coefficient are also important for study because they strongly influence the laser–metal interaction [14]. Additionally, the influence of the environment on the interaction is important (for example, laser irradiation in different gas environments) because many metals tend to form an oxide and other compounds’ layers on the surface, which may have an impact on the optical properties of metals as well as the increase in absorption of laser irradiation [15,16,17].

The change in the surface’s morphology is often accompanied by the formation of specific surface structures such as craters, capillary waves, hydrodynamic scattering, periodic structures, etc. [18,19,20,21,22]. There are several structure formation mechanisms in the molten phase and their transition to the solid phase by solidification. One of these mechanisms occurs during the metal irradiation by laser where laser fluence is near the threshold and has a low pulse number. In this way, the structures in the form of parallel waves form on the surface target that repeats in periods with a value corresponding to the laser wavelength. These structures are called laser-induced periodic surface structures (LIPSS) [23,24]. LIPSS strongly depend on the pulse number, e.g., exposition time, but also depend on other laser processing parameters. The formula for calculating the LIPSS periods, according to the most accepted plasmon combination theory, is:

(1) $$\Lambda_{LIPSS}=\frac{\lambda }{\eta \pm sin\theta }$$

where *η* = R [ε_m_ε_d_/(ε_d_ + ε_m_)]^1/2^ is a real part of effective refractive index for metal/air, *θ* is incident angle, *λ* is laser wavelength. ε_d_ is dielectric constant and ε_m_ is complex dielectric constant of a metal [25]. Thus, besides the pulse number, LIPSS also depend on laser wavelength, incident angle of laser beam and material properties. LIPSS have shown an impact on the optical properties of the metal surface, the increase of active surface, and biocompatibility [26].

In recent decades, a large amount of research has been devoted to the interaction of lasers with materials in different regimes [27,28,29,30]. Still, to our knowledge, no research has been dedicated to the interaction of lasers with nickel alloys in different atmospheres. Proposing the optimal irradiation conditions has a high significance for effective implementation of laser processing. It aims to ensure meeting the desired quality characteristics (i.e., process responses), thus improving efficiency and reducing time and resource consumption. The parametric optimization of the observed laser processing for different atmosphere conditions has not been explicitly studied in the literature. Various approaches have been carried out for the other laser-based processes, including the soft computing approaches [31,32,33,34] and the statistical approaches [35,36,37]. The soft computing approaches typically rely on neural models [34,38], whose development requires a large amount of data that are not always available from experimentations. Another issue refers to implementing metaheuristic algorithms, such as particle swarm optimization (PSO) [31,32,33,34], since their effectiveness highly depends on their inner parameter tuning. In other words, if the algorithm parameters are not properly selected, the algorithm converges to local optima [39]. This issue has not been explicitly addressed in the above studies that applied metaheuristics for optimizing different laser-based processes. The statistical approaches are mainly based on the response surface method (RSM) [35,36], the desirability function approach [37], and the Taguchi method. Although the RSM is one of the most frequently used methods to address multiple responses, it does not directly include the response variations. The model can be easily trapped into local optima for complex interrelationships characterized by a larger number of responses. The latter criticism is also applicable to the desirability function approach. In addition, both methods do not resolve correlations among responses [40]. The optimization method used in this study is based on Taguchi’s method and adequately addresses the shortcomings of the above statistical methods.

Surface modification effects following the interaction between 150 ps Nd:YAG laser light at 1064 nm wavelength and Nimonic 263, under various output pulse energies (6–30 mJ) and accumulated pulse number (10–2000), in the nitrogen-rich and argon-rich atmospheres, as well as the standard atmosphere conditions, are presented in this study. The argon- and nitrogen-rich atmospheres are chosen because of their inert gas nature. It is expected that the thermal effects would be reduced and more precise ablation would be achieved in the presence of these gases [41]. In addition, these gasses promote the formation of plasma that absorbs part of laser irradiation suitable for forming fine surface changes and structures [42,43]. Moreover, nitrogen can cause forming nitrides that have been proven to improve mechanical properties of alloys [44,45,46]. Electron scanning microscopy and profilometry determine the resulting changes in surface roughness, crater formation, and periodic surface structures. Changes in the chemical composition on the sample surface are analysed by energy-dispersive spectrometry. Effects of the process control parameters, pulse count, and pulse energy, on the output responses, surface roughness parameters, and ablation characteristics are also analysed by the statistical method PSO. LIPSS formation, with both high and low frequency periods, was detected in the nitrogen-rich and air atmospheres, and not detected in the argon-rich atmosphere. Oxide formation is dominant at the periphery of the modified surface. The most efficient ablation of Nimonic 263 surface, in regard to crater depth and increased roughness, is achieved in the argon-rich atmosphere, and optimal process parameters are determined.

## 2. Materials and Methods

The experiment is carried out on the group of Nimonic 263 samples with the dimension of each sample 100 × 30 × 2 mm. Prior to laser irradiation, samples were mechanically polished and cleaned. The irradiation is executed with picosecond Nd:YAG laser in standard atmospheric conditions i.e., in an air atmosphere (pressure of 1013 mbar and standard relative humidity) and the nitrogen- and argon-rich atmospheres. These gases were provided by a nozzle connected with a gas cylinder and flow meter (flow rate around 1 L/min) and located in front of the sample surface. This experiment used a quartz lens with a focal length of 170 mm. The stand-off distance, i.e., the distance between the sample and lens, is the same as the focal length (170 mm) to achieve the most effective ablation. Given that the distribution of a laser beam is Gaussian, the laser beam energy is lower on the periphery of the sample than at the centre. Mode TEM_00_, in which the laser operates, gives the output laser beam a circular shape. The conditions and parameters of laser irradiation are given in Table 1.

Changes caused by picosecond Nd:YAG laser interaction with Nimonic 263 superalloy in different environmental conditions are analysed by optical microscope EPITZP 2 (Carl Zeiss, Jena, Germany), scanning electron microscope JSM-6610LV SEM (JEOL, Tokyo, Japan) with energy-dispersive spectrometer X-Max Large Area Analytical Silicon Drift EDS. Contact stylus profilometry (Talysurf Series 2 profilometer, Taylor-Hobson Ltd., Digisurf software, Leicester, UK) is used for surface topography characterization: *Ra*, average surface roughness [47], *rms*, root mean square average of profile height deviations from the mean line [47], *PV*—peak to valley [47]; ablation depth measurements, two-dimensional profiles, and three-dimensional surface topography maps. The resolution was 0.25 μm in *x*-direction, 1 μm in *y*-direction, and 3 nm in *z*-direction. Filtering and evaluation process was done in accordance with ISO 25178 Surface Texture (Areal Roughness Measurement) international standard. The results obtained from profilometry analysis were processed in Origin program (version 9.0) with the polynomial curve fit of second order.

Additional experiments were conducted to investigate effects of the process control parameters (number of accumulated pulses—N_p_, pulse energy—E_p_) on the output responses: (I) *R_a_*, (II) *rms*, (III) ablation rate, *AR*, drilling depth per laser pulse; (IV) presence of craters or crater formation, *Cr*, bulk material removal; (V) circularity, *C*, the ration between max diameter of spot and min diameter of spot [48]; and (VI) PV, for the experimental conditions that were estimated to induce the most precise modification and efficient ablation, based on surface characterization results.

## 3. Results and discussion

### 3.1. Damage Threshold Fluence, F_th_, and Heat-Affected Zone, HAZ

The damage threshold fluence (F_th_), which represents the minimum laser energy fluence that causes visible changes on the sample surface [13,29], was determined for the interaction of Nimonic 263 superalloy surface with picosecond laser irradiation at different pulse energy values. The effect which picosecond laser irradiation causes on the surface of Nimonic 263 superalloy was determined by the amount of incident laser irradiation absorbed by the material and the surface parameters. Further energy transfer is described by the HAZ parameter, which represents the heat-affected zone. Considering the TEM_00_ mode of the picosecond laser irradiation regime, the threshold fluences are determined based on the dependence of the diameter of the laser irradiation spot in relation to the logarithmic value of the pulse energy and by drawing the best line from which the value for the diameter of the laser beam, ω_0_, is obtained [49,50]. The value of ω_0_ is to calculate damage threshold fluence, F_th_. For the interaction between Nd:YAG picosecond laser irradiation, wavelength 1064 nm, and pulse duration 150 ps, with Nimonic 263 superalloy surface, the following values of damage threshold fluences are calculated: 2.2 J cm^−2^ for the standard atmospheric conditions, 3.6 J cm^−2^ for the nitrogen-rich atmosphere, and 2.8 J cm^−2^ for the argon-rich atmosphere.

After the absorption of laser irradiation, and if it is assumed that the thermal capacity and thermal conductivity are constant, the resulting heat wave extends to the distance:

(2) $$l_{th}\approx \sqrt{D\times \tau }$$

where *D* (cm^2^s^−1^) is thermal diffusivity, *τ* (s) is duration length of the laser pulse, and *l_th_* (μm) is the length of thermal diffusivity i.e., heat-affected zone (HAZ) [51,52]. Under given experimental conditions, the calculated theoretical value of HAZ is ~22 nm. Theoretically, HAZ is independent of the external atmosphere, since it includes only material parameters and laser pulse duration.

### 3.2. Characterization of Surface Effects and Profilometry Analysis

**Figure 1:**
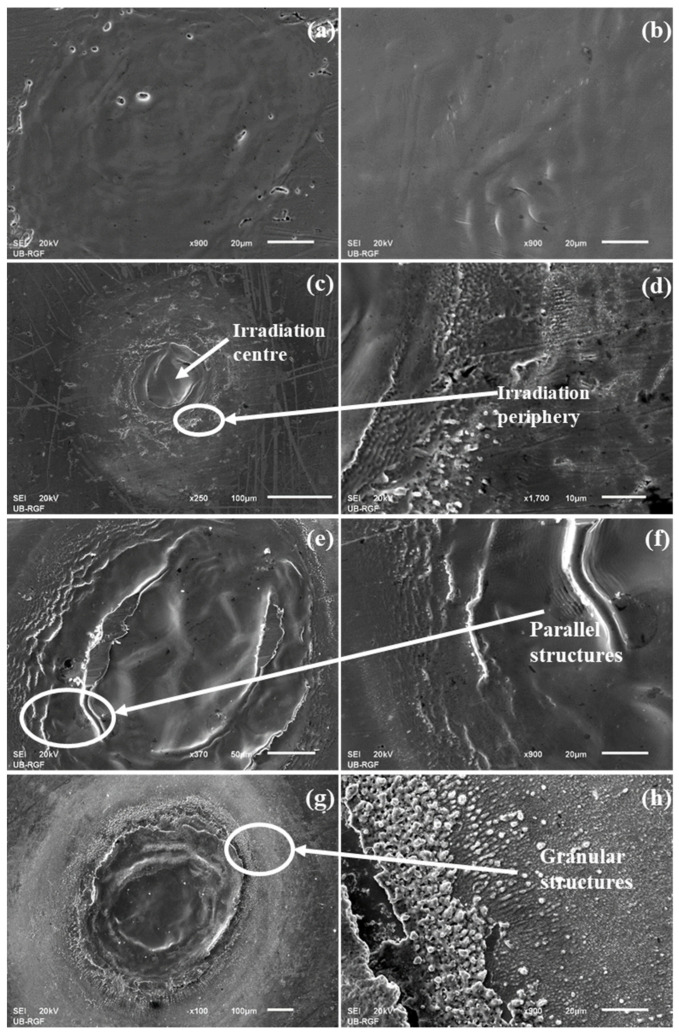
SEM microphotographs of Nimonic 263 superalloy surface after the irradiation by picosecond Nd:YAG laser, wavelength 1064 nm, pulse duration 150 ps: (**a**) whole spot and (**b**) periphery, N_p_ = 10, F = 5.50 J cm^−2^, SA conditions; (**c**) whole spot and (**d**) periphery, N_p_ = 100, F = 5.50 J cm^−2^, SA conditions; (**e**) whole spot and (**f**) periphery, N_p_ = 1000, F = 15.1 J cm^−2^, AA conditions; (**g**) whole spot and (**h**) periphery, N_p_ = 1000, F = 19.1 J cm^−2^, NA conditions.

After the irradiation of Nimonic 263 surface by ten accumulated pulses, the melting of the material surface occurred due to the absorption of the incident irradiation without any significant loss of material/ablation; surface changes are pronounced by an increased waviness in the centre of the irradiation sample due to rapid solidification of the molten material, as shown for SA conditions (Figure 1a,b). After ten accumulated pulses, the change in the atmospheric conditions and pulse energy leads to the similar surface effects of the Nimonic 263 superalloy, which can be described as “gentle” ablation.

Theoretically, and for the pulse duration below 5 ps, the lattice temperature and electron temperature in the metallic sample are different, and the bulk material heating occurs after the pulse is finished, which enables precise ablation [53,54]. However, in the case of increment of accumulated laser pulses and laser pulse duration of 150 ps, even though they do not fall into the nanosecond regime, pronounced melting occurs, the precise modification/ablation of the metallic surface is more difficult to achieve. Increasing the accumulated picosecond laser pulses leads to more prominent effects on the metallic surface, primarily hydrodynamic features such as resolidified expelled molten material at the rims of the spot, resolidified droplets, and distinguished craters [55,56,57].

Considering the Gaussian distribution of the laser beam, with the pulse count of more than ten, there is a distinct and clear transition between centre of the irradiation target and the periphery and between the periphery of laser-modified and the unmodified surface of Nimonic 263 superalloy, Figure 1. After 100 accumulated pulses, there is a more prominent melting of the material and its expulsion to the target periphery (Figure 1c,d). The formation of relatively shallow craters occurs, with a depth of up to ~10 μm (Table 2). There is also the “puckering” and progression of the molten material to the periphery of the irradiated target sample. It can be said that the ablation of the material is not so efficient, and the redistribution of the material is more dominant. With 1000 accumulated pulses, the appearance of the granular structures is present (Figure 1g,h) with an average diameter of ~0.8 μm in SA conditions (F = 5.50 J cm^−2^) and ~3.1 μm in NA conditions (F = 19.1 J cm^−2^). In AA conditions (F = 15.1 J cm^−2^), no granular structures are present, but there is a formation of parallel structures with the value of periods around 1.2 μm (Figure 1e,f). As for the craters, they have formed in the centre of the laser modified surface of the Nimonic 263 superalloy.

**Figure 2:**
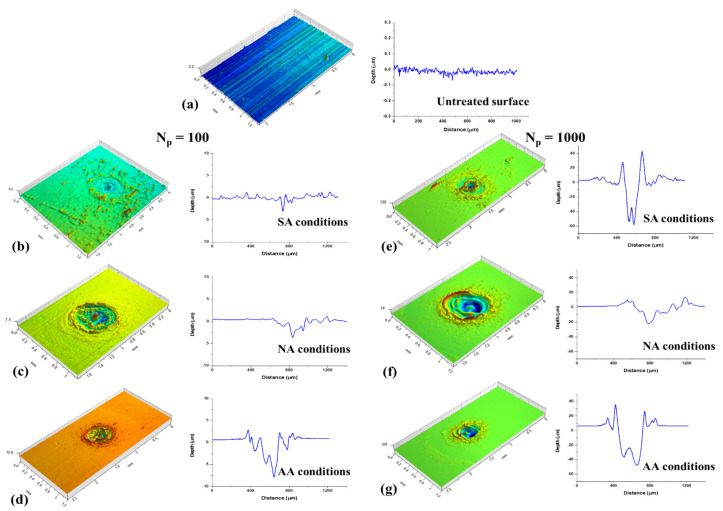
2D and 3D profiles of an untreated and laser treated Nimonic 263 surface under different environmental conditions and with different numbers of accumulated pulses after the irradiation with laser pulse energy E_p_ = 30 mJ; (**a**) represents 2D and 3D profile of an untreated surface; (**b**–**d**) represent 2D and 3D profiles of laser treated surface with 100 accumulated pulses; (**e**–**g**) represent 2D and 3D profiles of laser treated surface with 1000 accumulated pulses. Different environmental conditions are given in 2D profiles.

Profilometry analysis was used to examine the depth of traces obtained by laser irradiation, and the surface roughness parameters of the Nimonic 263 superalloy. Figure 2a presents the profilometry analysis performed at base Nimonic 263 material. Figure 2b–d present topography of surfaces irradiated by 100 accumulated pulses in SA, NA, and AA conditions, respectively. Figure 2e–g present topography of surfaces irradiated by 1000 accumulated pulses in SA, NA, and AA conditions, respectively.

With ten accumulated pulses, as can be seen from SEM microphotographs, regardless of the pulse energies and atmospheric conditions, there are shallow dents caused by laser irradiation with an average depth of up to ~3 μm (Table 2). After 100 and more applied pulses, as expected, there is an increase in the ablation depth at all pulse energies. The surface effects are comparable since there is only increased roughness and shallow craters, which can be attributed to the rearrangement of molten material, which confirms the SEM conclusions. Considering relatively low fluence values, the main process can be considered as ‘gentle’ ablation. Further increasing the pulse count leads to more prominent ablation and pronounced craters, as presented for laser action of 1000 accumulated pulses at lowest fluence values, Table 2. Craters are characterized by accumulated resolidified molten material at the rims and apparently at the bottom of the craters due to the insufficient energy density for more efficient material removal or evaporation, especially in the argon-rich atmosphere. At the lowest pulse energy (E_p_ = 6 mJ), the highest value of depth is obtained in AA conditions (~61.2 μm, F = 6.10 J cm^−2^) with 2000 accumulated pulses (Table 2). At higher values of pulse energies, E_p_ = 15 mJ and E_p_ = 30 mJ, the highest values of ablation depths are also obtained in the argon-rich atmosphere: ~123.8 μm (F = 15.1 J cm^−2^) and ~92.2 μm (F = 30.3 J cm^−2^). In SA conditions, after 1000 accumulated pulses, the ablation depth appears to reach saturation at the highest pulse energy. It can be said that the argon-rich atmosphere is more favourable for the formation of craters than the standard atmospheric conditions and the nitrogen-rich atmosphere. This potentially happens because air and nitrogen have higher thermal conductivity than argon [58], increasing cooling rates in SA and NA conditions than in AA conditions, thus providing more efficient ablation [59]. Regarding the dependence of the ablation depth from pulse count at different values of laser fluences, and under the same environmental conditions, for example, the argon-rich atmosphere, the results imply that the ablation depth increases up to 2000 accumulated pulses at the 15.2 J cm^−2^ and 30.3 J cm^−2^ fluences. It reaches saturation of ~56 μm ablation depth at 1000 pulses with a laser fluence of 6.10 J cm^−2^.

**Figure 3:**
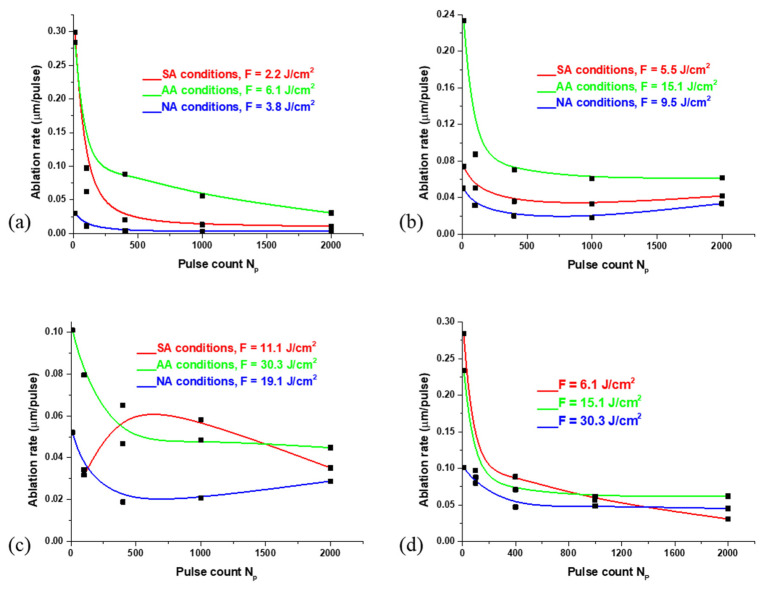
The dependence of ablation rate on the pulse count ranging from 10 to 2000 accumulated pulses, after the irradiation of the Nimonic 263 superalloy surface by picosecond Nd:YAG laser in different environmental conditions and with pulse energy of: (**a**) E_p_= 6 mJ; (**b**) E_p_ = 15 mJ; (**c**) E_p_ = 30 mJ. In (**d**), the dependence is given with the same environmental conditions (argon-rich atmosphere) and different laser fluences.

The following parameter obtained from profilometry analysis results is the ablation rate which represents the thickness of the ejected layer of material per laser pulse, shown in Figure 3. With the lowest applied pulse energy (E_p_ = 6 mJ), in SA conditions, the ablation rate is 0.30 μm/pulse after ten accumulated pulses (Figure 3a). A similar situation happens in AA conditions. In contrast, in NA conditions, the ablation rate after ten pulses is 0.03 μm/pulse. With the increase in the number of pulses, the ablation rate decreases; after 1000 accumulated pulses, in SA and AA conditions, the ablation rate is 0.01 and 0.03 μm/pulse, respectively, while in NA conditions, the value of the ablation rate is below 0.01 μm/pulse. It can be said that the ablation effect is dominant when the number of accumulated pulses is lower (N_p_ < 100). In contrast, after 100 and more pulses, the hydrodynamic effects, in the form of melting and accumulation of material in the target centre, are more pronounced. With a pulse energy of 15 mJ, the ablation rate has a similar trend as the lowest pulse energy. In AA conditions, the highest value of ablation rate (0.23 μm/pulse) is obtained after ten accumulated pulses, while in SA and NA conditions the values of ablation rate are lower by factors 3–4 (0.07 and 0.05 μm/pulse, respectively). With the increase in pulse number, the ablation rate decreases the most in NA conditions, and the least in AA conditions (Figure 3b). After the highest applied pulse energy (E_p_ = 30 mJ), in AA and NA conditions, the ablation rate values decrease with an increase in pulse count: from 0.10 to 0.04 μm/pulse and from 0.05 to 0.03 μm/pulse, respectively (Figure 3c). With this pulse energy, the ablation rate under standard atmospheric conditions has a different trend: the value of ablation rate decreases from 10 to 100 accumulated pulses, but after 100 pulses, the ablation rate increases by a factor of 2, so in SA conditions, it is possible to achieve efficient ablation even with a large number of accumulated pulses. Generally, higher values of the ablation rate in AA conditions are in accordance with previous studies regarding the laser ablation of graphite in the argon atmosphere [60].

**Figure 4:**
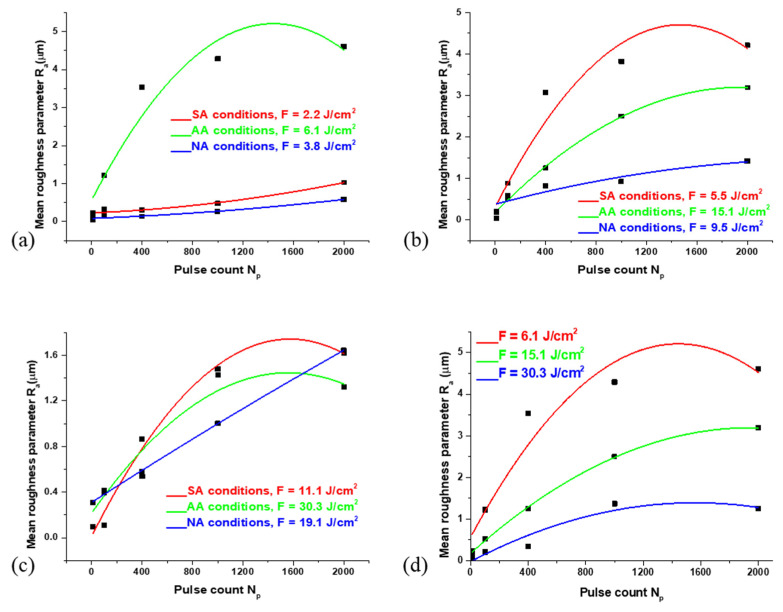
The dependence of mean roughness parameter R_a_ on the pulse count ranging from 10 to 2000 accumulated pulses, after the irradiation of the Nimonic 263 superalloy surface by picosecond Nd:YAG laser in different environmental conditions and with pulse energy of: (**a**) E_p_ = 6 mJ; (**b**) E_p_ = 15 mJ; (**c**) E_p_ = 30 mJ. In (**d**), the dependence is given with the same environmental conditions (argon-rich atmosphere) and different laser energy fluences.

Figure 4 represents the dependence of the surface roughness parameter R_a_ on the number of accumulated pulses. The following can be summarized: with the lowest pulse energy of 6 mJ applied, a similar tendency in increasing the value of surface roughness parameter R_a_ have Nimonic 263 samples which are irradiated in SA and NA conditions, with laser energy fluences of 2.20 and 3.80 J cm^−2^, respectively (Figure 4a), even after 2000 accumulated pulses, while in AA conditions (F = 6.10 J cm^−2^) the value of surface roughness parameter decreases after 1000 applied pulses. However, it should be noted that the surface roughness parameter values are highest in AA conditions (R_a_ = 4.61 μm) and the lowest in NA conditions. When a pulse energy of 15 mJ is applied (Figure 4b), the value of parameter Ra decreases with an increase in pulse count. With this pulse energy, the highest value of parameter R_a_ is achieved in SA conditions after 2000 accumulated pulses (R_a_ = 4.20 µm, F = 5.50 J cm^−2^). Generally, the lowest values of parameter R_a_ are achieved with the highest pulse energy (Figure 4c), E_p_ = 30 mJ, with the highest values of R_a_ in SA and NA conditions (R_a_ ≈ 1.62 µm, F = 11.1 J cm^−2^, and F = 19.1 J cm^−2^, respectively).

After the complete SEM and profilometry analysis, it can be noted that the most efficient ablation, regarding Nimonic 263 superalloy surface irradiated with picosecond Nd:YAG laser, occurs in the argon-rich atmosphere. In summary, after ten accumulated pulses, values of ablation depth in AA conditions are higher for a factor 3 and 2 than in SA conditions for the pulse energies values of 15 mJ and 30 mJ, respectively. For the lowest applied pulse energy (E_p_ = 6 mJ), values of ablation depth are similar. After 1000 accumulated pulses, values in AA conditions are higher for factors 4, 2, and 1.2 than in SA conditions, for the pulse energy values of 6 mJ, 15 mJ, and 30 mJ, respectively. With regard to the efficient ablation in the argon-rich atmosphere, these environmental conditions are chosen for process parametric optimization.

### 3.3. Laser-Induced Perodic Surface Structures (LIPSS)

**Figure 5:**
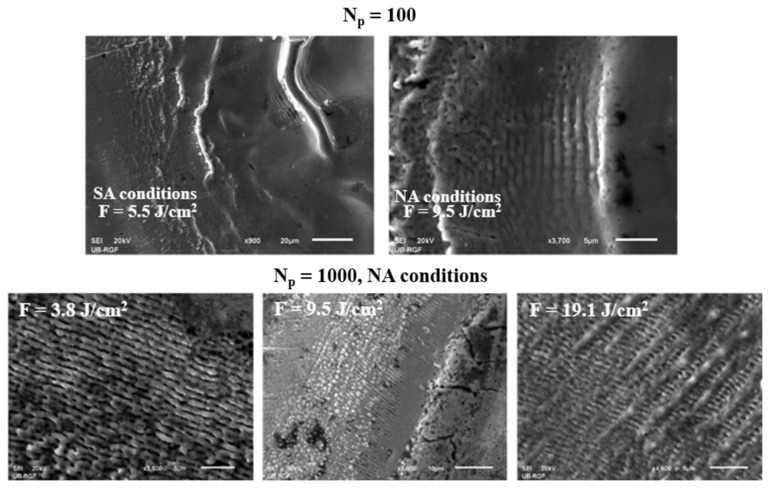
LIPSS structures obtained in standard atmospheric conditions and nitrogen-rich atmosphere after the irradiation of Nimonic 263 superalloy surface by picosecond Nd:YAG laser.

After the picosecond laser irradiation in SA and NA conditions, laser-induced periodic surface structures (LIPSS) were formed with 100 and 1000 applied pulses (Figure 5). With 100 accumulated pulses, the period values of LIPSS structures are close to the laser wavelength (1064 nm), namely 1040 nm and 1051 nm, in SA conditions (F = 5.50 J cm^−2^) and NA conditions (F = 9.50 J cm^−2^), respectively. Analysing the SEM microphotographs, it can be concluded that, after 1000 accumulated pulses, LIPSS structures are the most prominent in NA conditions (Figure 5). They appeared on the periphery of the irradiated target at all fluence values, while in SA conditions, they are present at 5.50 J cm^−2^ with a period value of ~984 nm. As for their period values in NA conditions, some are close to the laser wavelength (Λ ≈ 937 nm, F = 3.80 J cm^−2^) while others are significantly below laser wavelength: 435 nm (F = 3.80 J cm^−2^), 755 nm (F = 9.50 J cm^−2^) and 717 nm (F = 19.1 J cm^−2^). According to the theory of LIPSS formation and period values [24,61,62], this indicates a formation of both low-spatial frequency (LSFL, Λ~λ) and high-spatial frequency (HSFL, Λ << λ) LIPSS structures. The mechanism of LIPSS formation is a complex process and has been a subject of research for many years. One of the most accepted mechanisms of LIPSS formation is that these structures are formed by interference of incident laser irradiation and surface plasmons [25]. The appearance of LIPSS is expected after the interaction of low-fluence short-pulse laser irradiation (order of picoseconds and femtoseconds) with metallic surfaces under standard atmospheric conditions, as well as in the presence of gases such as nitrogen, helium, argon, etc. [63,64]. It can be seen from SEM microphotographs that there is a formation of net-like LIPSS structures, i.e., structures oriented parallel and perpendicular to the laser beam polarization (Figure 5). The formation of both types of LIPSS is confirmed in NA conditions, at fluence value of 3.80 J cm^−2^ and with 1000 accumulated pulses. In the AA conditions, no LIPSS structures are observed. This phenomenon can be explained by the fact that argon decreases the surface tension of the material melted by laser irradiation, allowing deeper penetration, thus preventing the formation of fine surface structures such as LIPSS [59]. Additionally, after the irradiation with 2000 accumulated pulses, no LIPSS were generated in any environmental conditions. This is expected, since this number of accumulated pulses causes intense melting of the target due to extended exposure to the laser irradiation.

### 3.4. EDS Analysis

Table 3 presents the results of the semiquantitative EDS analysis. A contribution of each element is given in the form of a mass percentage (wt%). EDS analysis was carried out on several locations of the target surface, including the target’s centre and periphery. Regarding the chemical composition of the Nimonic 263 surface before the laser irradiation, it can be noticed that the state of elements (of which the superalloy is composed) is in a preserved chemical ratio (Ni:Cr:Co:Mo ≈ 50:20:20:6, with other elements in a total of ~4 wt%). After the modification in standard atmospheric conditions, oxygen appears on the periphery of a modified target with a mass fraction of 11 wt%. In NA and AA conditions, oxygen appears only on the periphery of a modified target with a higher mass fraction: ~14 and 15 wt%, respectively. It is evident that oxidation on the superalloy surface is probably a dominant chemical process. The oxidation of the periphery of the target surface led to the mass fraction decreasing of the most abundant elements in the superalloy: nickel and cobalt. In contrast, the mass fraction of chromium and molybdenum stayed almost the same (decreasing up to 1 wt%). When increasing the pulse energy and changing the number of accumulated pulses, there were no significant changes in the chemical composition of the Nimonic 263 superalloy surface. However, semi-quantitative EDS analysis was used only for the estimation of the elemental composition on the Nimonic 263 surface, and the main conclusions are derived based on the comparison of the non-irradiated area and the laser-modified surface areas. Therefore, they are formulated as the proposed, not definite, explanations.

Oxide formation on the surface of metallic materials is desirable because many metals tend to form oxide layers on the surface which can impact optical properties of materials and increase the absorption of laser irradiation, as well as positively impact the biocompatibility of implants and improve the material corrosion resistance [16,65].

### 3.5. Process Parametric Optimization

Investigating effects of the process control parameters on the output responses is done for the laser modification in argon experimental conditions as they are estimated as the most precise modification and efficient ablation, based on the analysis presented in Section 3.1 and Section 3.2.

Table 4 presents experimental results for the argon-rich atmospheres. Three measurements were performed for each response (i.e., the sample size equals three), and the average values are shown in Table 4.

The optimization approach relies on the Taguchi’s robust parameter design and, specifically, on the Taguchi’s quality loss (QL) function that explicitly indicates a level of the user’s dissatisfaction when using a product whose characteristic (i.e., process response) deviates from the desired value. This is especially important for multi-response processes, since QL stipulates the relative importance of the product characteristics (i.e., process responses) for the user [40]. The QL calculation relies on the signal to noise ratio (SNR) that addresses both the response mean and its variation, using formulas proposed by G. Taguchi [66]. This computation depends on the type of a response according to the SNR analysis that recognizes three types of responses: (i) smaller the better (STB) type, where the objective is to minimize the response value; (ii) nominal the best (NTB) type, where the aim is to achieve a predefined (nominal) vale; and (iii) larger the better (LTB) type, where the response value needs to be maximized.

For the observed process, the responses R_a_, rms, AR, and PV are of the LTB type, while responses C_r_ and C belong to the NTB type (the target, i.e., nominal value for both responses equals 1).

Desired responses depend on the targeted application of the sample material. Thus, the crater formation, significant ablation rate, and circularity are desirable for drilling purposes, like controlling the hole/crater quality, since Nimonic 263 is a superalloy with a superior hardness that is generally challenging for micromachining. On the other hand, the increased surface roughness responses, R_a_, rms, and PV can be favourable for surface patterning [67,68], for example, which could enable efficient lubrication, inducing periodic structures which cause the altering of the optical properties, increased wettability, etc.

The obtained QLs are normalized with respect to the maximal and minimal values for all experimental runs, and for each response separately. The normalized QL values (NQL ∈ [0, 1]) are listed in Table 5 for the argon-rich experimental conditions.

In order to address correlations among responses, principal component analysis (PCA) was carried out on NQLs to obtain a set of uncorrelated data for further processing (*j* is the number of principal components, *k* is the experimental run numbers, *i* is the response number, and *V_ij_* are the eigenvector’s elements) [40]:

(3) $$Y_{j}\left(k\right)=\overset{p}{\underset{i=1}{\sum }}NQLF_{i}\left(k\right)\cdot V_{ij}$$

On the obtained scores presented in Table 5, grey relational analysis (GRA) was carried out using the procedure specified in [69]. The absolute values of scores are standardized and transformed into grey relational coefficient $\varepsilon_{j}\left(k\right)$. The gray relational grade, proposed as a single process performance, was developed by averaging $\varepsilon_{j}\left(k\right)$ and using weights *w_j_* obtained from PCA:

(4) $$\gamma_{k}=\overset{P}{\underset{j=1}{\sum }}w_{j}\varepsilon_{j}\left(k\right)$$

The resulting process performances ($\gamma_{k}$ ∈ [0, 1]) for the argon-rich atmosphere conditions are given in Table 5, calculated based on the weights from PCA: [0.608, 0.212, 0.099, 0.067, 0.013, 0.000]. By involving all principal components in an impartial manner, the total response variance is included, and the process performance is formed in a fully objective way. The larger the process performance, the more superior the process in terms of meeting requirements for multiple responses.

Finally, the effects of control parameters are computing by averaging the process performance across the parameter levels, where level 1, 2, and 3 refers to the minimal, middle, and maximal value of the control parameter used in the experiment, respectively. The parameter values that rise the process performance are taken as optimal. From Table 6, it can be seen that the following settings are obtained as optimal: Np = 1000, Ep = 15 mJ for the argon-rich atmosphere.

**Figure 6:**
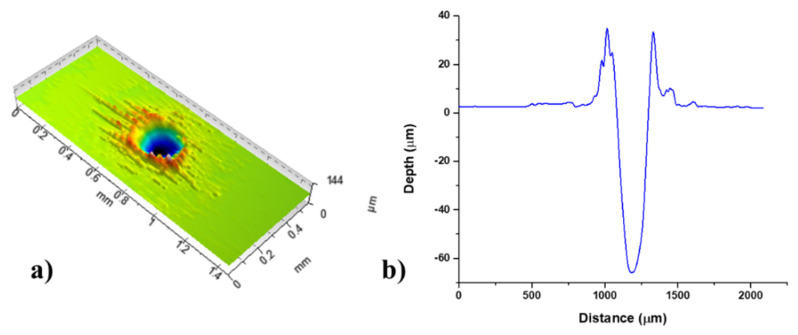
Nimonic 263 area modified by F = 15.1 J cm^−2^, pulse count 1000 in the argon-rich atmosphere: (**a**) 3D map, (**b**) 2D profile.

Figure 6a presents the 3D profile of the modified area/spot achieved by the optimal parameters: laser action of 1000 accumulated pulses, and fluence valued 15.1 J cm^−2^ in the argon-rich atmosphere. By visual observation of the presented profile, it could be seen that the crater shape is nearly circular with well-defined rims. The roughness is relatively high and uniformly distributed around the hole. The surface is nicely defined and suitable for adhesion of lubricates or other intended agents. Two-dimensional profiles, presented in Figure 6b, show the regular shape of a crater, with clear-cut inner walls and the absence of unexpelled molten material. Therefore, the condition of adopted parameters are favourable for meeting the desired responses for the argon-rich atmosphere.

## 4. Conclusions

In this experiment, Nimonic 263 samples were irradiated by picosecond Nd:YAG laser light. Surface laser modification was done by changing laser parameters and under different environmental conditions. The following can be concluded:

- The laser modification with ten accumulated pulses, in all environmental conditions, can be characterized as “gentle” ablation;
- After the irradiation with 100 and more accumulated pulses, hydrodynamic effects occur, such as melting of the material and its partial expulsion to the periphery of the target with the formation of relatively shallow craters; a part of the material after melting accumulates in the centre of the target due to rapid cooling;
- Efficient ablation occurs with 1000 applied pulses in all atmospheres, especially at medium (E_p_ = 15 mJ) and high (E_p_ = 30 mJ) values of applied pulse energy; the most pronounced craters, and therefore the most efficient ablation, were obtained in the argon-rich atmosphere (the depth value of 123.8 μm);
- The values of surface roughness parameter R_a_ are highest in AA and SA conditions while being the lowest in NA conditions;
- LIPSS are observed after 100 accumulated pulses in SA and NA conditions, while at 1000 accumulated pulses, LIPSS structures are more pronounced in the nitrogen-rich atmosphere with the formation of both LF-LIPSS and HF-LIPSS;
- EDS analysis shows potential oxide formation located at the periphery of the irradiated surface;
- With the assessment from SEM and profilometry analysis that the argon-rich atmosphere is best for precise modification and efficient ablation, and after the process parametric optimization has been done, the following laser parameters are obtained as optimal: E_p_ = 15 mJ and N_p_ = 1000.

## Figures and Tables

**Table 1:** The conditions and parameters of Nd:YAG picosecond laser irradiation in the interaction with Nimonic 263 superalloy.

Laser	Nd:YAG
Wavelength	1064 nm
Pulse duration	150 ps
Number of accumulated pulses (N_p_)	10
	100
	400
	1000
	2000
Pulse energy (mJ)	6.0 ± 0.5
	15 ± 2
	30 ± 3
Atmosphere conditions	Standard atmospheric conditions (SA)
	Nitrogen-rich atmosphere (NA)
	Argon-rich atmosphere (AA)
Fluences (J cm^−2^)	SA: 2.20; 5.50; 11.1
	NA: 3.80; 9.50; 19.1
	AA: 6.10; 15.1; 30.3

**Table 2:** Values of ablation depth after the irradiation of Nimonic 263 surface with picosecond laser by changing the laser parameters and environmental conditions.

Laser Energy, E_p_ (mJ)	Pulse Count, N_p_	Ablation Depth (µm)		
		SA Conditions	AA Conditions	NA Conditions
		*F = 2.20 J cm^−2^*	*F = 6.10 J cm^−2^*	*F = 3.80 J cm^−2^*
6	10	2.99	2.84	0.30
	100	6.24	9.71	1.13
	400	8.29	35.34	1.72
	1000	13.32	55.98	3.48
	2000	21.81	61.19	6.68
		*F = 5.50 J cm^−2^*	*F = 15.1 J cm^−2^*	*F = 9.50 J cm^−2^*
15	10	0.74	2.34	0.50
	100	5.08	8.76	3.14
	400	14.39	28.33	8.04
	1000	32.84	61.29	17.97
	2000	83.65	123.79	66.80
		*F = 11.1 J cm^−2^*	*F = 30.3 J cm^−2^*	*F = 19.1 J cm^−2^*
30	10	0.50	1.01	0.52
	100	3.17	7.95	3.42
	400	26.08	18.69	7.49
	1000	58.10	48.40	20.60
	2000	70.28	89.50	57.41

**Table 3:** EDS analysis of Nimonic 263 superalloy surface before and after the picosecond laser irradiation under different experimental conditions.

Elemental Composition (wt%)										
Elements		O	Al	Si	Ti	Cr	Fe	Co	Ni	Mo
**Unmodified surface**			0.54	0.23	1.71	19.9	0.52	20.1	50.1	6.85
**AA conditions,** **E_P_ = 6 mJ,** **N_P_ = 100**	centre		0.42	0.20	1.60	18.4	0.64	20.7	50.8	7.24
	periphery	14.4	0.59	0.35	2.07	18.0	0.66	16.4	42.1	5.34
**SA conditions,** **E_P_ = 15 mJ,** **N_P_ = 100**	centre		0.48	0.28	1.81	19.6	0.58	20.4	50.7	6.22
	periphery	11.3	0.51	0.29	1.57	19.1	0.51	17.4	43.7	5.62
**NA conditions,** **E_P_ = 30 mJ,** **N_P_ = 100**	centre		0.44	0.17	0.81	17.9	0.65	21.4	53.3	5.25
	periphery	13.7	0.64	0.39	2.25	19.9	0.46	16.8	40.3	5.53

**Table 4:** Experimental observations for argon-enriched atmosphere.

	Control Parameters		Output Responses					
*Exp. no.*	N_p_	E_p_ (mJ)	R_a_ (µm)	Rms (µm)	AR (µm/Pulse)	Cr	C	PV (µm)
*1*	10	6	0.06	0.08	0.030	0	0.77	0.63
*2*	10	15	0.21	0.28	0.050	0	0.87	2.18
*3*	10	30	0.08	0.12	0.052	0	0.68	1.26
*4*	100	6	0.15	0.21	0.011	0	0.87	1.69
*5*	100	15	0.21	1.11	0.031	1	0.84	6.57
*6*	100	30	0.08	0.27	0.034	1	0.77	2.58
*7*	1000	6	0.27	0.38	0.003	1	0.87	4.24
*8*	1000	15	0.93	1.31	0.018	1	0.84	13.8
*9*	1000	30	0.88	1.32	0.021	1	0.82	14.33

**Table 5:** Normalized quality loss (NQL) values, principal components scores, and process performance for argon-rich atmosphere.

*Exp. no.*	NQL Ra	NQL rms	NQL AS	NQL Cr	NQL C	NQL PV	Y1	Y2	Y3	Y4	Y5	Y6	Process Performance
*1*	1.000	1.000	0.007	1.000	0.421	1.000	1.981	−0.462	−0.049	0.147	−0.130	0.005	0.457312
*2*	0.075	0.077	0.000	1.000	0.000	0.082	0.462	−0.472	0.360	0.649	−0.174	−0.024	0.604226
*3*	0.547	0.454	0.000	1.000	1.000	0.248	1.325	−0.124	0.876	0.153	−0.059	0.001	0.520357
*4*	0.151	0.142	0.071	1.000	0.000	0.137	0.549	−0.535	0.326	0.580	−0.188	−0.010	0.576900
*5*	0.075	0.001	0.006	0.000	0.102	0.007	0.076	0.038	0.063	−0.061	−0.028	−0.015	0.956374
*6*	0.547	0.084	0.004	0.000	0.421	0.058	0.491	0.182	0.203	−0.320	−0.263	−0.008	0.673881
*7*	0.044	0.040	1.000	0.000	0.000	0.020	−0.078	−0.789	0.166	−0.589	0.025	0.024	0.765962
*8*	0.000	0.000	0.024	0.000	0.102	0.000	0.033	0.018	0.079	−0.047	0.030	−0.017	0.991870
*9*	0.000	0.000	0.017	0.000	0.181	0.000	0.063	0.053	0.135	−0.069	0.051	−0.030	0.939569

**Table 6:** Effects of process control parameters on process performance for argon-rich atmosphere.

Process Control Parameters	Np	Ep
Level 1	0.5273	0.6001
Level 2	0.7357	0.85083
Level 3	0.8991	0.7113

## Data Availability

Not applicable.
